# Prognostic Factors for Open Globe Injuries and Correlation of Ocular Trauma Score in Tianjin, China

**DOI:** 10.1155/2015/345764

**Published:** 2015-09-29

**Authors:** Yu Meng, Hua Yan

**Affiliations:** Department of Ophthalmology, Tianjin Medical University General Hospital, No. 154, Anshan Road, Tianjin 300052, China

## Abstract

*Purpose*. To investigate prognostic factors that influence the final visual acuity (VA) and to correlate the ocular trauma score (OTS) with the final VA in open globe injuries. *Methods*. A retrospective review of 298 patients with open globe injuries admitted to Tianjin Medical University General Hospital was carried out from January 1, 2010, till December 31, 2014. Prognostic factors influencing the final VA in patients with open globe injuries and the correlation between OTS and the final VA were examined. *Results*. Three hundred and fourteen eyes from 298 patients with open globe injuries were analyzed. Males had a higher rate of open globe injury than females (83.56% versus 16.44%). Mean age was 45.46 ± 17.48 years (5–95 years). In a univariate analysis, prognostic factors influencing the final VA included initial VA, relative afferent papillary defect (RAPD), vitreous hemorrhage, lens injury, endophthalmitis, hyphema, retinal detachment, and the zone of injury. In a multiple logistic regression analysis, initial VA, RAPD, and the zone of injury were considered to be independent risk factors. The OTS correlated with final VA (*r* = 0.988, *p* = 0.000). *Conclusion*. In our study, the most important prognostic factors influencing the final VA were initial VA, RAPD, and the zone of injury. The OTS was of great importance for patients and ophthalmologists.

## 1. Introduction

Open globe injury, defined as a full thickness wound of the eye wall [1], is a major but preventable cause of permanent visual impairment and blindness in the world [2]. The World Health Organization program estimated that approximately 750,000 cases of ocular trauma are hospitalized per year, and 200,000 cases are open globe injuries worldwide [3].

Despite advances in ophthalmic surgery such as operating microscopes, vitreoretinal techniques, and surgical skills together with improvements in the awareness of visual prognosis, instrumentations, and other factors that have led to better outcomes, there remain a number of eyes that cannot be salvaged [4]. They impact not only the individuals, but also the country's healthcare system [5].

Based on literature review, factors likely to predict the final visual acuity (VA) after open globe injury are initial VA, mechanism or type of injury, zone of injury, adnexal trauma, relative afferent pupillary defect (RAPD), retinal detachment, uveal or retinal tissue prolapse, vitreous hemorrhage, lens injury, hyphema, delay to surgery, and number of operative procedures [6–24]. One of the most important uses of knowing about prognostic factors is that it helps the physician in counselling the patient and his family and preparing him for the outcome.

Ocular trauma score (OTS) system suggested by Kuhn et al. [25] is to predict the final VA after an open globe injury. Kuhn et al. [25] analyzed more than 2500 injured eyes from the United States and Hungarian Eye Injury Registries (USEIR) and evaluated more than 100 variables with the goal of identifying specific predictors. OTS is calculated by assigning definite numerical raw points to six variables: initial VA, rupture, endophthalmitis, perforating injury, retinal detachment, and RAPD (Table 1). The scores are stratified into five categories that give the predictabilities of final VA.

Little data is currently available on open globe injury in Tianjin. The aims of this study were to determine prognostic factors influencing the final VA and to validate the OTS in patients with open globe injuries.

## 2. Methods

A retrospective review of medical records of all consecutive patients with open globe injuries from January 1, 2010, till December 31, 2014, at Tianjin Medical University General Hospital was carried out.

Case notes were examined to determine demographic data (age and gender), eye(s) involved, cause and place of injury, and type of injury. Initial VA, zone of injury, and clinical signs (hyphema, lens injury, RAPD, endophthalmitis, retinal detachment, and vitreous hemorrhage) were recorded. Management, follow-up data, duration of hospitalization, injury time (from the point of injury to presentation at the clinic), and final VA were also documented. We also utilized the OTS to evaluate the final VA.

Type of injury was based on the Ocular Trauma Classification Group: rupture, penetrating injury, intraocular foreign body, or perforating injury [1]. Zone of injury was defined according to the Ocular Trauma Classification Group: zone 1 (the whole cornea, including corneoscleral limbus), zone 2 (corneoscleral limbus to a point 5 mm posterior into the sclera), and zone 3 (posterior to the anterior 5 mm of the sclera) [1].

Initial and final VAs were classified as no light perception (NLP) and light perception (LP)/hand motion (HM), 1/200–19/200, 20/200–20/50, and ≥20/40. A good visual outcome was defined as a final VA of 20/200 or better, while a poor visual outcome was defined as a final VA of less than 20/200.

Patients with previous ocular surgery and preexisting ocular conditions affecting VA as well as those with less than 6 months of follow-up were excluded.

Statistical analysis was carried out using SPSS version 19.0 statistical software (IBM, Armonk, NY, USA). Data was expressed as the mean ± SD (Standard Deviation) for continuous variables. Univariate logistic regression analysis was used to examine the association between prognostic factors (type of injury, initial VA, zone of injury, and clinical signs) and the final VA. Furthermore, all the factors found significant in univariate logistic analysis were included in the multivariate analysis. The odds ratio (OR) and 95% confidence interval (CI) for variables were calculated as well. A *p* value of 0.05 was considered statistically significant.

## 3. Results

This current study included data from 314 eyes from 298 patients over a 5-year period. Two hundred and forty-nine (83.56%) patients were males and 49 (16.44%) patients were females. Mean age was 45.46 ± 17.48 years (5–95 years). One hundred and sixty-five (55.03%) patients occurred in aged 21–50-year-old group. Right eyes were associated with 135 (45.30%) patients and left eyes with 147 (49.33%) patients. Sixteen (5.4%) patients had bilateral eyes involvement. Mean duration of hospitalization was 15.01 ± 11.73 days (2–68 days). Two hundred (69.46%) patients took less than 24 hours to look for medical care after their injuries; however, 9 (14.9%) patients still took more than 4 days. Mean duration of follow-up was 8.40 ± 2.30 months (6.10–10.70 months). One hundred and forty-nine (50.00%) injuries happened in the workplace, 77 (25.84%) happened at home, and 53 (17.78%) on the road. Most of the injuries were caused by metallic objects (153, 51.34%) followed by traffic accidents (56, 18.79%) (Table 2).

Regarding type of injury, penetrating injury (192, 61.15%) accounted for the majority of open globe injuries, followed by intraocular foreign body (83, 26.43%). Rupture (17, 5.41%) and perforating injury (22, 7.01%) accounted for the remaining open globe injuries. Of the 22 perforating injury eyes, 7 (31.82%) eyes had final VA of less than 20/200, and 15 (68.18%) eyes had final VA of 20/200 or better. All patients received intravitreal and systemic antibiotics. Two hundred (90.40%) eyes underwent one surgical procedure, 94 (29.91%) eyes underwent two surgical procedures, and 20 (6.40%) eyes underwent three or more surgical procedures (Table 2).

In terms of the zone of injury, 170 (54.14%) eyes had zone 1 injuries, 84 (26.75%) eyes had zone 2 injuries, and 60 (19.11%) eyes had zone 3 injuries. Hyphema was associated with 215 (68.47%) eyes and vitreous hemorrhage was associated with 112 (35.67%) eyes. Lens injury was found in 112 (35.67%) eyes. RAPD was noted in 47 eyes (14.97%). Endophthalmitis was present in 36 eyes (11.46%). Retinal detachment was observed in 32 (10.19%) eyes (Table 3).

**Figure 1:**
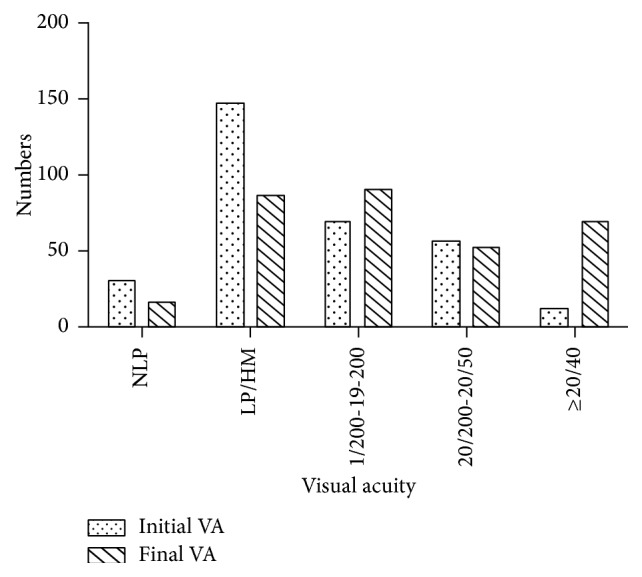


The distribution of initial and final VA was illustrated in Figure 1. Twelve (3.8%) eyes had initial VA of 20/40 or better, 56 (17.8%) eyes had initial VA of 20/200–20/50, 69 (22.0%) eyes had initial VA of 1/200–19/200, 147 (46.8%) eyes had initial VA of LP/HM, and the remaining 30 (9.6%) eyes had initial VA of NLP. After about 6 months follow-up, 70 (22.29%) eyes had VA of 20/40 or better, 52 (16.57%) eyes had final VA of 20/200–20/50, 90 (28.66%) had final VA of 1/200–19/200, 86 (27.39%) had final VA of LP/HM, and the remaining 16 (5.10%) eyes had final VA of NLP.

Based on the univariate logistic regression analysis, prognostic factors such as initial VA (*p* = 0.000), RAPD (*p* = 0.002), retinal detachment (*p* = 0.002), vitreous hemorrhage (*p* = 0.000), hyphema (*p* = 0.000), lens injury (*p* = 0.000), endophthalmitis (*p* = 0.014), and zone of injury (*p* = 0.000) adversely affected the final VA (Table 3).

All factors found significant in univariate logistic analysis were included in the multivariate logistic analysis to further evaluate their associations with final VA. Initial VA (*p* = 0.000, OR = 8.329, 95% CI = 3.310–20.959), RAPD (*p* = 0.023, OR = 4.788, 95% CI = 1.241–18.478), and the zone of injury (*p* = 0.000, OR = 2.709, 95% CI = 1.577–4.653) were found to be the most statistically significant for the final VA (Table 4).

Three hundred and fourteen eyes were classified within OTS categories one through five. Against USEIR-OTS system, our study had a smaller sample size; we still could see close resemblance between the scores in our study and that in USEIR study of OTS. The OTS correlated with final VA (*r* = 0.988, *p* = 0.000) (Table 5).

## 4. Discussion

We found that open globe injuries occurred predominantly in males, consistent with other studies [13, 26]. This might be due to gender-based behavior and male involvement in higher risk of working activities. Mean age in our study was 45.46 ± 17.48 years and most of the injuries occurred in groups aged 21–50 which were similar to other studies [13, 27]. Most of the patients (50.00%) occurred in the workplace. Better education of workers as regards workplace safety and the provision and use of protective eye wear will help reduce the incidence of open globe injury in the workplace. Majority of the patients (69.46%) could seek for medical care timely. However, nine (14.9%) patients still took more than 4 days, and all of them ended up with final VA of less than 20/200, 6 cases with a result of final VA of LP/HM, and 3 cases with a result of final VA of 1/200–19/200. Poverty and a lack of awareness might hamper timely management of ocular injuries.

In our study, of the 30 eyes with initial VA of NLP, 14 eyes ended with improved vision, and the remaining 16 eyes still had VA of NLP at last follow-up. Among the 14 eyes that ended with improved vision, only 1 eye regained useful ambulatory vision; the remaining 13 eyes achieved final VA of less than 20/200. For the 16 eyes with final VA of NLP, 4 eyes were as a result of primary enucleation, 6 eyes were as a result of secondary enucleation, and 6 eyes were as a result of phthisis bulbi. Schmidt et al. [22] have demonstrated that initial VA was found to correlate significantly with the final VA in open globe injuries. Our study showed similar results that patients who had initial VA of 20/200 or better had improvement in final VA; however, majority of patients with initial VA of LP/HM or worse had poor final VA. Based on multivariate logistic regression analysis, initial VA had statistically significant influence on the final VA (*p* < 0.001).

Pieramici et al. [19] found that if RAPD was present, final VA was significantly worse. In our study, 39 patients had final VA of less than 20/200 if RAPD was present; using multivariate logistic regression analysis, presence of RAPD had statistically significant influence on the final VA (*p* = 0.023). However, it was a concern that there was a tendency not to examine the pupil responses during the initial examination of open globe injuries, so examining for a reverse afferent should be instructed as an essential part.

Retinal detachment, induced by direct trauma or traction of proliferative vitreous in open globe injuries, was found to be a significant prognostic factor by Hutton and Fuller [28] and Thompson et al. [29]. When it occurs, photoreceptor cells are probably seriously injured and may lead to limited final VA. In our study, 29 (90.63%) patients with retinal detachment had poor final VA of less than 20/200, confirming its importance as a prognostic factor (*p* < 0.001) by multivariate logistic regression analysis.

Vitreous hemorrhage, caused by rupture of blood vessels in the ciliary body, retina, urea, or sclera, was found to be a prognostic factor [30]. When it occurs, it may be related to serious damage of eye tissues. In our study, 92 (60.92%) patients with vitreous hemorrhage had final VAs that were less than 20/200. Using univariate logistic regression analysis, presence of vitreous hemorrhage had statistically significant influence on the final VA (*p* = 0.000).

Hyphema also played a role in final VA [31]. Madhusudhan et al. [26] found that patients who did not have hyphema were twice less likely to have the final VA of less than 3/60 compared with patients having hyphema. Our study also showed similar results that eyes with hyphema were not prone to achieve a good final VA of 20/200 or better (*p* = 0.000).

Lens injury, caused by direct lesion or the development of cataract, was also an important factor of the final VA [32]. In our study, 72 (64.29%) eyes had final VA of less than 20/200 if lens injury was present (*p* = 0.000). However, Tök et al. [15] found that lens injury had no effect on the final VA because of its association with zone 1, the possibility of performing lens surgery quickly after injury, and improvements in cataract surgery and lens technology.

Endophthalmitis has been mentioned as a prognostic indicator by Williams et al. [33]. Endophthalmitis is associated with special spectrum of organisms such as* Bacillus* and* Staphylococcus* and* Streptococcus* species [34]. In our study, endophthalmitis developed in 36 (11.0%) eyes. Among them, 29 (80.56%) eyes had poor final VA of less than 20/200, and 7 (19.44%) eyes had good final VA of 20/200 or better. The association between endophthalmitis and the final VA in our study was also statistically significant (*p* = 0.014).

Hutton and Fuller [28] found that wounds involving zone 2 or 3 resulted in significantly higher rates of poor final VA than those involving zone 1 in open globe injuries. Similarly, Madhusudhan et al. [26] also found that patients whose wounds involve zone 3 had 20 times the risk of having poor final VA when compared with those whose wounds involve zone 1. This could be explained by the fact that posterior wounds could cause irreparable damage to photoreceptors such as retina and optic nerve; despite anatomic correction, final VA might remain limited [35]. By multivariate analysis in our study, zone of injury was a significant predictor influencing the final VA (*p* = 0.000).

OTS study [25] stated that a patient with OTS category one will have a higher risk of poorer final VA as against a patient with OTS category five who will have a higher probability of better final VA. In our study, we found that only 16.98% of patients with OTS category one had final VA of 20/200 or better, whereas 30.19% of patients with OTS category one had final VA of NLP. Of the patients with OTS category five, 100% had final VA of 20/40 or better. Another study by Man and Steel [35] also suggested that OTS possibly had predictive value of the final VA in open globe injury. OTS is of great importance for patients and ophthalmologists.

Several limitations of our study should be acknowledged and discussed. First, as a hospital-based study, we identified a small part of all open globe injuries in Tianjin, and a nationwide eye injury surveillance system should be established. Second, it was related to insufficient medical records such as lids and adnexal injury, extent of injury, and zone involvement intraoperatively, but those data could not be included in the statistical analyses. Third, whether delayed presentation or high risk mechanism injury is related to infection rate and ultimately visual outcome or not was not analyzed. Despite those limitations, we still identified several parameters as potential predictive factors.

In conclusion, prognostic factors for the final VA included initial VA, RAPD, vitreous hemorrhage, lens injury, endophthalmitis, hyphema, retinal detachment, and the zone of injury. However, initial VA, RAPD, and the zone of injury, as independent risk factors, were the most important recommendation for further consideration. OTS, a very comprehensive score to predict final VA in patients with open globe injuries, should be more commonly used by ophthalmologists of the world.

## Figures and Tables

**Table 1:** Calculating the ocular trauma score (OTS): variables and raw points.

Variables	Raw points
Initial VA	
NLP	60
LP/HM	70
1/200–19/200	80
20/200–20/50	90
≥20/40	100
Rupture	−23
Endophthalmitis	−17
Perforating injury	−14
Retinal detachment	−11
RAPD	−10

**Table 2:** Characteristics of patients with open globe injuries.

Variables	*n*
Total patients (total injured eyes)	298 (314)
Female/male	49/249
Age (years, mean ± SD^a^)	45.46 ± 17.48
Right/left/both	135/147/16
Mean duration of hospitalization (days)	15.01 ± 11.73
Injury time (hours)	
0–24	200
≥24	98
Places of injuries	
Workplace	149
Home	77
School	4
Road	56
Others	12
Cause of injuries	
Metallic objects	153
Traffic accidents	56
Falling	44
Blunt objects	28
Others	17
Diagnosis	
Penetrating injury	192
Intraocular foreign body	83
Perforating injury	22
Rupture	17

^a^SD: standard deviation.

**Table 3:** Univariate logistic regression analysis of factors which affected final visual acuity.

Variables	Final VA		*p*
	≥20/200	<20/200	
Type of injury			0.8000
Penetrating injury	76	116	
Perforating injury	15	7	
Intraocular foreign body	26	57	
Rupture	5	12	
Initial VA			0.000
≥20/200	61	185	
<20/200	61	7	
RAPD			0.002
No	114	153	
Yes	8	39	
Retinal detachment			0.002
No	119	163	
Yes	3	29	
Vitreous hemorrhage			0.000
No	102	100	
Yes	20	92	
Hyphema			0.000
No	57	42	
Yes	65	150	
Lens injury			0.000
No	102	200	
Yes	40	72	
Endophthalmitis			0.014
No	115	163	
Yes	7	29	
Zone of injury			0.000
1	90	80	
2	32	52	
3	0	60	

**Table 4:** Multiple logistic regression analysis of factors which mostly affected final visual acuity.

Variables	*p*	OR	95% CI
Initial VA	0.000	8.329	3.310–20.959
RAPD	0.023	4.788	1.241–18.478
Zone of injury	0.000	2.709	1.577–4.653

**Table 5:** Percentage with final VA and OTS categorical distribution in this study and the OTS study.

Raw OTS	OTS	NLP	LP/HM	1/200–20/200	20/200–20/50	≥20/40
0–44	1	30/74	53/15	17/7	0/3	0/1
45–65	2	0/27	46/26	51/18	3/15	0/15
66–80	3	0/2	0/11	17/15	38/31	45/41
81–91	4	0/1	0/2	0/3	44/22	56/73
92–100	5	0/0	0/1	0/1	0/5	100/94

This study/OTS study.
